# Artificial intelligence technology in aortic valve disease: a decade of scientometric and narrative review

**DOI:** 10.3389/fcvm.2026.1843658

**Published:** 2026-07-09

**Authors:** Peng Hei, He Ren, Wenshuai Ma, Wei Fang, Yan Li

**Affiliations:** 1 Department of Cardiology, Tangdu Hospital, The Fourth Military Medical University, Xi’an, Shaanxi, China; 2 Cardiac Rehabilitation Center, Department of Cardiology, Tangdu Hospital, The Fourth Military Medical University, Xi’an, Shaanxi, China

**Keywords:** aortic valve, artificial intelligence, bibliometrics, citespace, VOSviewer

## Abstract

**Background:**

Aortic valve disease, particularly aortic stenosis, poses a growing global health burden with aging populations. Artificial intelligence technology offers promising tools for diagnosis, risk stratification, and prognosis prediction, yet the knowledge structure of this interdisciplinary field remains unsystematically characterized.

**Objective:**

This study aims to conduct a scientometric analysis to delineate the research landscape, identify hotspots, and trace evolutionary trends of AI technology applications in aortic valve disease over the past decade.

**Methods:**

We retrieved relevant literature published between January 2016 and January 2026 from the Web of Science Core Collection and Scopus databases. After screening, 270 eligible articles were included. CiteSpace and VOSviewer were employed to perform visualization analyses of authors, institutions, countries, journals, keywords, and co-citation networks.

**Results:**

Annual publications increased steadily, with the United States leading in both output and influence. The Mayo Clinic emerged as the most prolific institution. Research hotspots focused on AI-assisted diagnosis, risk stratification, and prognosis prediction for aortic stenosis, primarily using deep learning and machine learning techniques. Keyword clustering revealed themes spanning disease diagnosis, therapeutic technologies, AI-enabled applications, and clinical outcomes. Co-citation analysis highlighted key studies on AI-enhanced electrocardiography and echocardiography for valve disease detection.

**Conclusions:**

AI technology research in aortic valve disease is advancing rapidly. Based on the keyword clustering and timeline analysis, we propose a conceptual mapping of AI techniques onto clinical phases. Future efforts should prioritize developing multimodal models, facilitating clinical integration, and enhancing patient lifecycle management.

## 1 Introduction

Aortic valve diseases primarily include aortic stenosis (AS) and aortic regurgitation (AR). Among these, AS is a common cardiac condition in the elderly population, with its incidence gradually increasing with age. In Western countries, the prevalence of AS is approximately 2.0% in individuals aged ≥65 years and approximately 4% in those aged ≥85 years (1). AR exhibits a lower prevalence than AS: 0.6% vs. 1.4% in the 65–74 age group, and 1.7% vs. 4.6% in those ≥75 years old (2). With accelerating population aging, the clinical burden of aortic valve disease continues to grow, emerging as a significant global public health issue (3). Traditional diagnosis and treatment primarily rely on imaging assessments such as echocardiography, computed tomography (CT), and cardiac magnetic resonance imaging (MRI), combined with clinical guidelines for decision-making (4). However, the field still faces numerous challenges, including difficulty in identifying the early asymptomatic stage, lacking of precise models for predicting disease progression, individual variations in determining the optimal timing for surgical intervention, and an incomplete long-term postoperative follow-up system. These issues drive ongoing exploration for more efficient and precise auxiliary tools in this field.

In recent years, AI technologies, particularly machine learning and deep learning, have demonstrated significant potential in medical image analysis, disease risk stratification, prognosis prediction, and clinical decision support (5, 6). In the cardiovascular field, AI technology has been preliminarily applied to the intelligent diagnosis and management of coronary artery disease, heart failure, and arrhythmias (7–9). Concurrently, studies have utilized AI technology for automated valve calcification assessment and regurgitation quantification analysis in ultrasound or CT images, as well as for optimizing risk models based on clinical data, demonstrating high accuracy and efficiency (10, 11).

Despite the gradual increase in related research, the knowledge structure and research hotspots in this interdisciplinary field lack systematic organization. Scientometric methods can reveal the knowledge foundation, frontier dynamics, and collaborative networks within a discipline through visualization and network analysis of literature data, providing objective evidence for future research directions (12). As mainstream scientific knowledge mapping tools, CiteSpace and VOSviewer have been successfully applied in multiple medical fields for research context deconstruction and trend prediction. However, no comprehensive scientometric analysis has been reported in the field of AI and aortic valve diseases.

Therefore, this study aims to employ CiteSpace and VOSviewer software to conduct a scientometric analysis of literature related to the application of AI technology in the field of aortic valve disease. By constructing co-occurrence maps of keywords, author collaboration networks, institutional networks, and co-citation networks of publications, it systematically deconstructs the research distribution, thematic evolution, and research hotspots within this domain. This analysis provides a reference framework for subsequent research and translational research.

## 2 Methods

### 2.1 Data sources

Search the Web of Science Core Collection (WoSCC) and Scopus databases (completed within one day to avoid bias from database updates), covering the time range from January 1, 2016, to January 10, 2026. The search strategy was developed following the PRISMA-S guidelines for literature searching. We combined controlled vocabulary (MeSH terms in WoSCC and Emtree terms in Scopus) with free-text keywords. see the supplement for the detailed search query.

**Figure 1:**
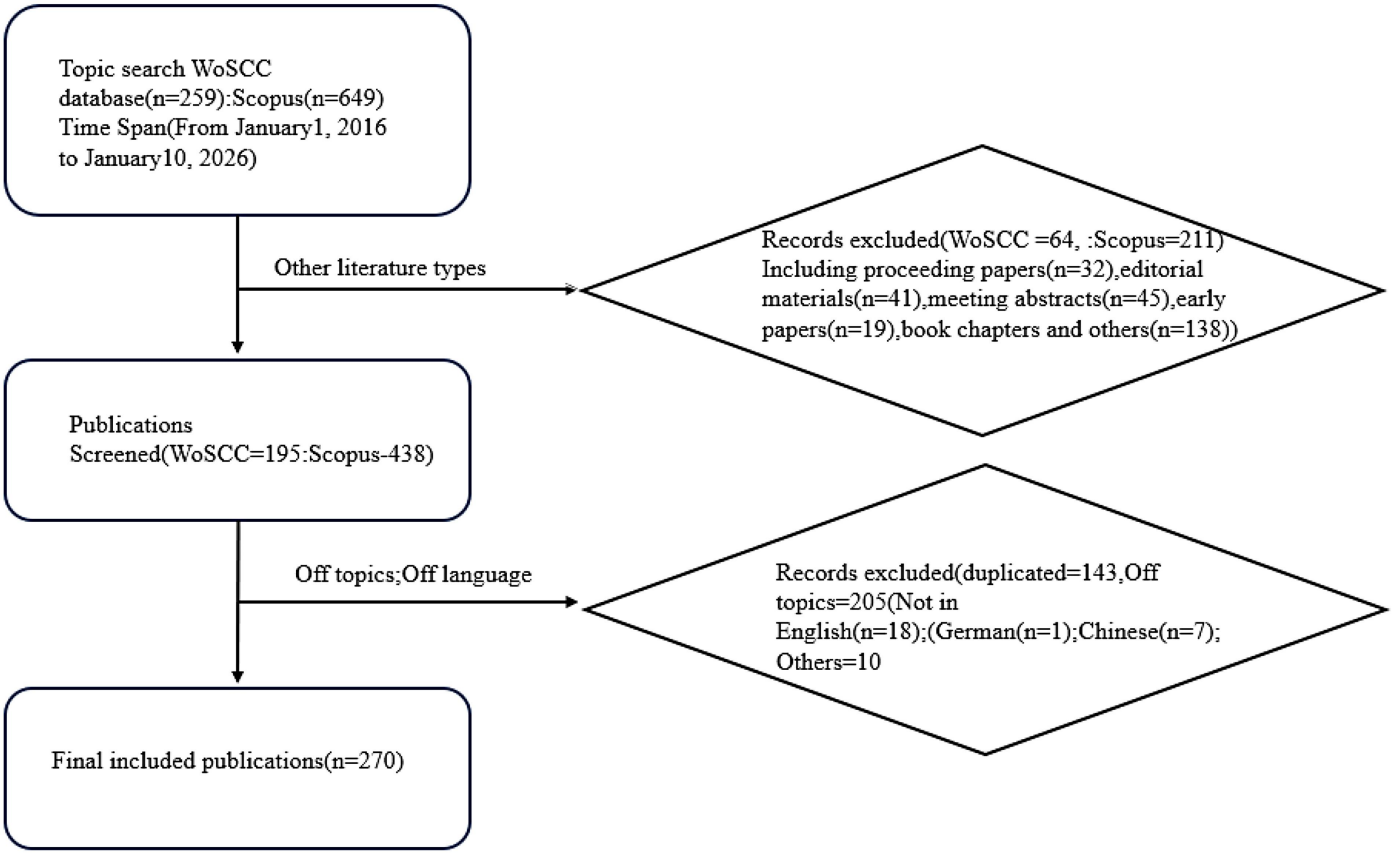
Literature screening flowchart.

A total of 908 documents were retrieved. Articles and review articles were selected, while conference abstracts and other relevant documents were excluded. Ultimately, 633 documents were imported into EndNote software. After removing irrelevant and duplicate documents, 270 eligible documents were included for bibliometric analysis (Figure 1).

### 2.2 Inclusion and exclusion criteria

Inclusion Criteria: Studies focusing on the application of artificial intelligence technology in aortic valve disease.

Exclusion Criteria: ① Duplicate publications; ② Articles with poor relevance to the research topic; ③ Article types such as theses, conference proceedings, books, newspapers, yearbooks, patents, etc.

### 2.3 Data processing

Two researchers independently extracted data, initially screening articles based on titles and abstracts, and systematically removing duplicates and irrelevant topics from the dataset. A secondary screening was conducted based on inclusion and exclusion criteria; any disputes were resolved through discussion with a third researcher. Information extracted from included studies comprised titles, authors, abstracts, publication years, institutions, and keywords.

WoSCC data is exported in Plain text file format. Select “Full Record and Cited References” for record content, and name the file “download_WOS.txt”; Scopus data is exported in Reference Manager (RIS) format, named “download_scopus.txt,” and imported into EndNote software. CiteSpace parameter settings: We used g-index (k = 1) to favor highly cited papers, default Top N (50)/Top N% (10%) to avoid arbitrary filtering (13), Pathfinder + pruning to simplify networks, and a minimum citation threshold of 5 for VOSviewer co-citation analysis, following standard practice (13, 27, 28).Use CiteSpace 6.3.1 with a time span from January 2016 to January 2026; Time slice: 1 year; Node types: Keywords, Authors, Research Institutions; Link strength: Cosine; Threshold: g-index (k = 1), Top N, Top N% (default values); Pruning methods: Pathfinder method, Pruning the merged network method. After parameter configuration, run the analysis to generate the corresponding knowledge map (13).

VOSviewer Parameter Settings: Using VOSviewer 1.6.18, select bibliographic coupling for journal co-occurrence analysis and cited references co-occurrence analysis; choose co-citation for reference co-citation analysis. Enable full counting. Adjust thresholds, attraction, repulsion, and node size as needed. Organize data and perform statistical analysis on retrieved bibliometric data using Microsoft Excel software, including generating charts depicting publication volume and growth trends. Adjust and optimize the clustering diagrams and network layouts generated by VOSviewer software using pajek64 portable 6.01 software.

### 2.4 Observation indicators

Based on the maps and data generated by CiteSpace and VOSviewer software, the primary indicators include: ① Spatio-temporal characteristics of literature, including the trend in English-language publications from January 1, 2016, to January 10, 2026; ② High-output authors and research institutions, along with collaboration patterns among countries; ③ Keyword co-occurrence networks; ④ The top 15 keywords by emergence intensity and their timeline maps.

## 3 Results

### 3.1 Analysis of publication volume

**Figure 2:**
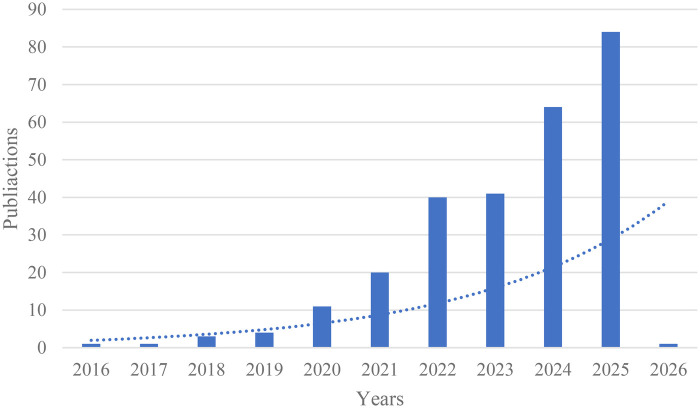
Annual publication volume based on the 270 included studies (2016–2026).

A total of 270 eligible articles were finally included in the analysis. Using CiteSpace to analyze annual publication volumes, the data was imported into Microsoft Excel to generate a line chart (Figure 2). From 2016 to 2026, the annual number of publications exhibited a consistent upward trend, with a notable acceleration starting in 2020. The highest annual output was observed in 2025 (84 papers), followed by 2024 (64 papers). Due to limitations in the search timeframe, it is anticipated that the upward trend in publications within this field will continue into 2026.

### 3.2 Co-occurrence analysis of authors

**Figure 3:**
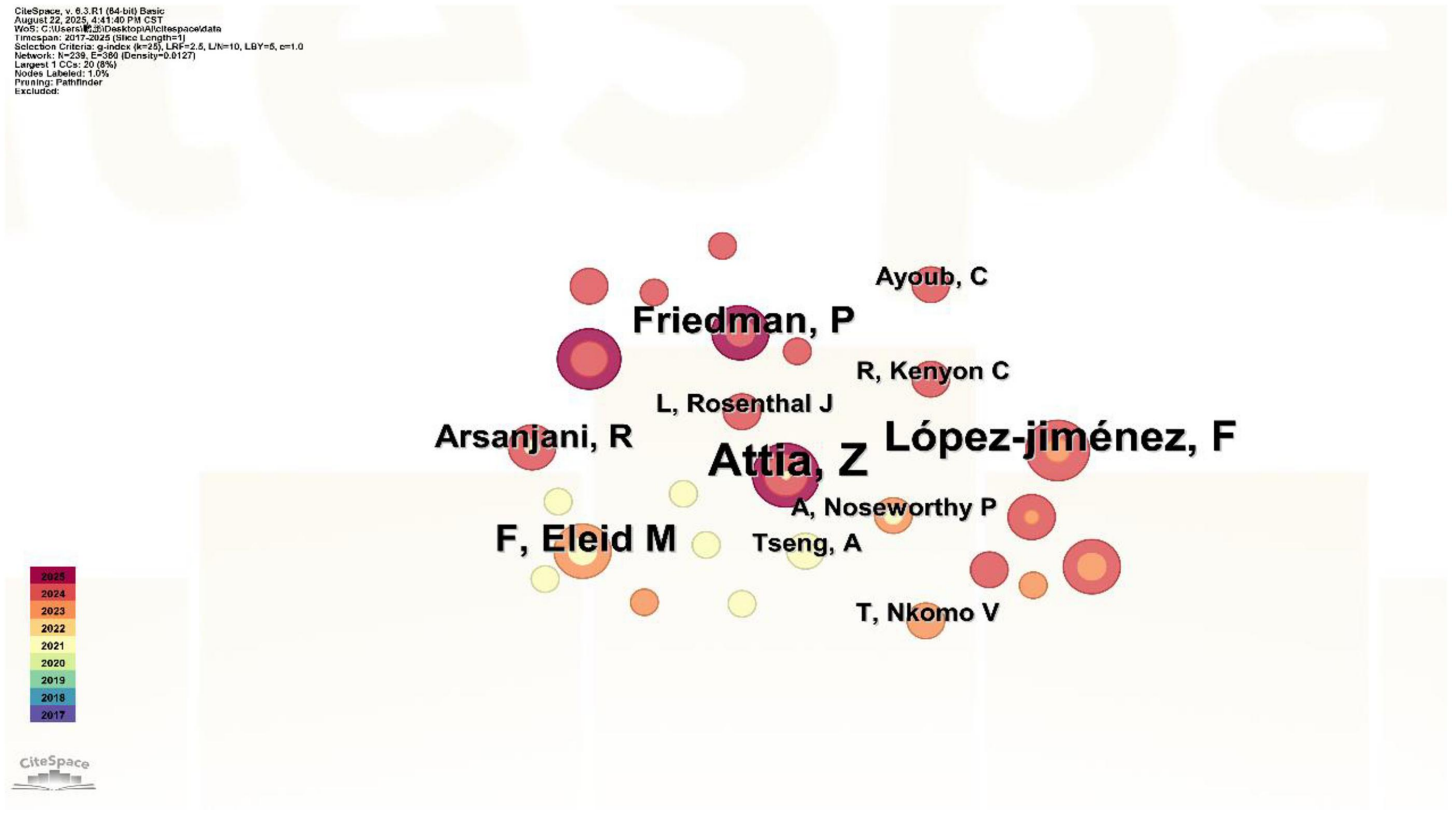
Co-occurrence map of authors in the application of AI technology. Node size = publication count; colors = clusters; lines = collaboration.

The co-occurrence network in this field includes 270 publications, involving 239 authors, with 360 connections and a density of 0.0127. According to Price's Law, the formula for calculating the publication volume of core authors is: M = 0.749 × √N_max_ (14), where N_max_ denotes the number of papers published by the author with the highest publication volume between 2016 and 2026. In this study, N_max_ = 6, and M ≈ 1.84. Thus, core authors are defined as those who published ≥2 papers. This study identified 80 core authors. The top 10 by publication volume are: Attia, Z (6), López-Jiménez, F (5), Li, Y (5), Hahn, Rebecca T (4), Eleid, M F (4), Friedman, P (4), Playford, D (4), Arsanjani, R (3), Prasad, Dasi Lakshmi (3), and A, Strange G (3). Currently, the most prolific author in this field is Attia, Z from the Mayo Clinic in Minnesota, USA. His team primarily focuses on AI-ECG, artificial intelligence-based electrocardiogram research, with extensive applications in diseases such as left bundle branch block, aortic valve stenosis, and hypertrophic cardiomyopathy (15–17). Close collaboration within the principal investigator teams has driven research and development in AI-ECG. The network density among teams exceeds 0.01, indicating varying degrees of cooperation between different groups. Strengthening and expanding such collaborations in the future is expected to further advance this field to higher levels (Figure 3).

### 3.3 Research countries and institutions analysis

#### 3.3.1 Country analysis

**Figure 4:**
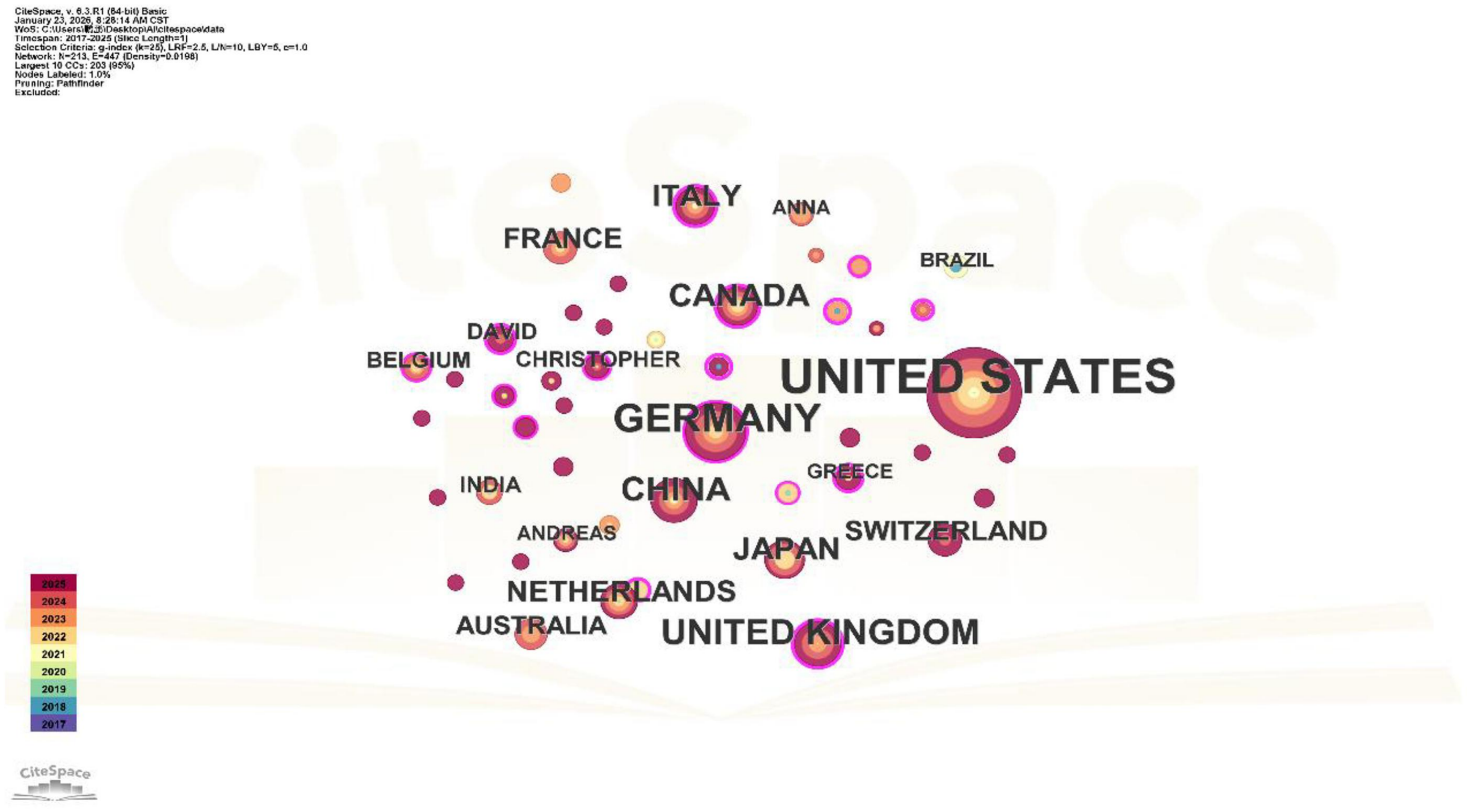
National co-occurrence map of AI technology research in aortic valve disease. Node size = publication volume; line thickness = collaboration strength.

Selecting “Country” as the node type, CiteSpace software was used to statistically analyze the countries publishing literature on AI technology in aortic valve disease research. The co-occurrence map of countries is shown in Figure 4. The country (or region) distribution map comprises 213 countries (or regions), with 447 collaborative connections and a network density of 0.0198. The number of connections between nodes indicates relatively frequent collaborative exchanges among countries. The top five countries by publication volume are: USA (116 papers); CHINA (31 papers); GERMANY (25 papers); UNITED KINGDOM (17 papers); CANADA (13 papers), with the USA significantly outpacing others. Analysis of node centrality reveals the top 5 countries are USA (0.91), JAPAN (0.60), GERMANY (0.42), GREECE (0.37), and SPAIN (0.29), indicating these nations maintain stronger connections with others (Figure 4, Table 1). Notably, among the top five countries by publication volume, only the USA and Germany achieved high centrality rankings, while others failed to enter the top 10 centrality list. This indicates that in this field, countries like China and Canada often confine their research domestically, neglecting transnational collaboration. Conversely, nations such as the USA and Germany exhibit high publication volumes and node centrality, demonstrating their deep involvement in pivotal collaborative projects within the field, thereby serving as intermediaries for knowledge exchange between different countries.

**Table 1:** Top 5 countries by publication volume on AI technology in aortic valve disease .

country (region)	publications	Centrality
USA	116	0.91
CHINA	31	0.06
GERMANY	25	0.42
UNITED KINGDOM	17	0.27
CANADA	13	0.21

#### 3.3.2 Institutional analysis

**Figure 5:**
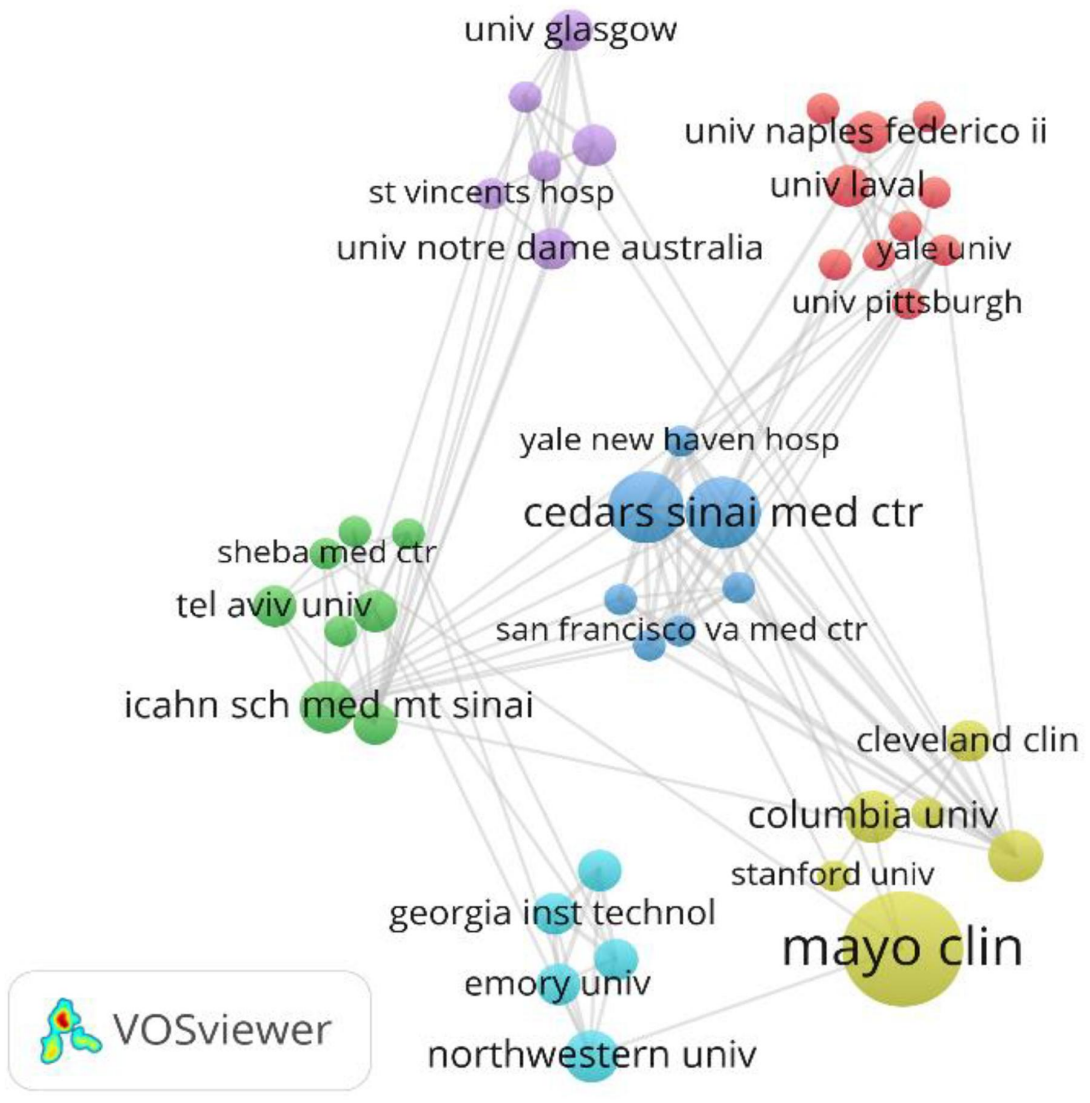
Institutions of AI technologies in the field of aortic valve disease. Node size = publication count; link thickness = collaboration intensity.

Using VOSviewer software, “Organizations” was set as the analysis unit to perform co-occurrence analysis on publishing institutions within this field (Figure 5, Table 2). This analysis included 212 institutions. The top five institutions by publication volume were: Mayo Clinic (11), University of California (6), Cedars-Sinai Medical Center (6), Yale School of Medicine (4), and Icahn School of Medicine (4). These institutions also occupy central positions within the collaboration network, demonstrating significant radiating and hub effects. In terms of citation frequency, the top five institutions were: Mayo Clinic (276), University of California (192), Cedars-Sinai Medical Center (166), Columbia University (125), and Yale School of Medicine (109). The data indicates that leading institutions in both publication volume and academic influence are concentrated in the United States, underscoring America's absolute dominance in this research field. It stands as the primary hub for knowledge production and collaboration in applying artificial intelligence technology to aortic valve disease research.

**Table 2:** Top 5 institutions by publication volume on AI technology in aortic valve disease.

Institution	publications	Citation frequency
Mayo Clinic	11	276
University of California	6	192
Cedars-Sinai Medical Center	6	166
Yale School of Medicine	4	109
Icahn School of Medicine	4	48

### 3.4 Journal visualization analysis

**Figure 6:**
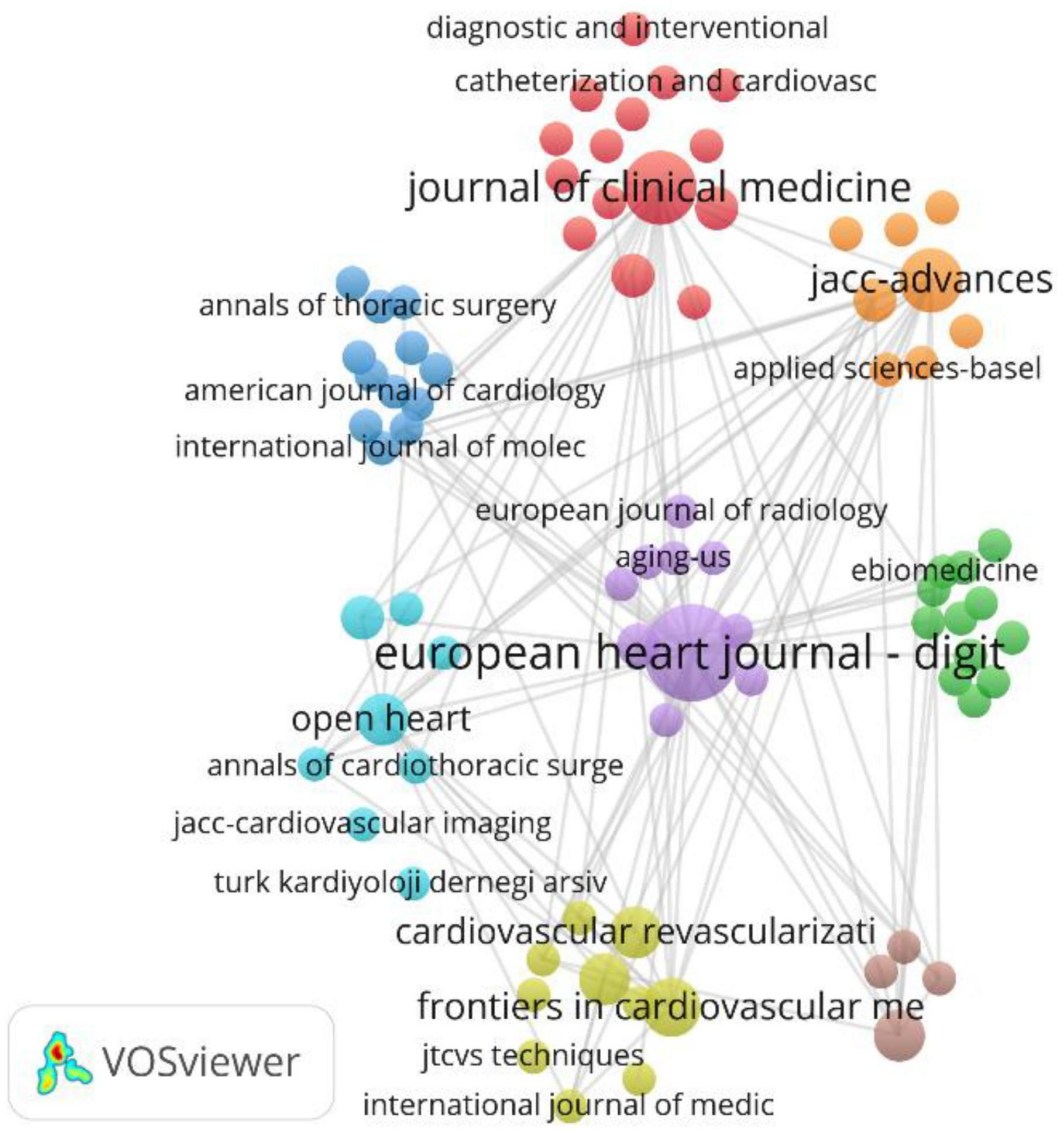
Co-occurrence Map of AI technology in research journals on aortic valve disease. Nodes = journals; colors = 8 thematic clusters.

Using the “bibliographic coupling-Sources” module in VOSviewer software, we conducted a visual analysis of journals publishing English-language literature in this research field. Pajek software was applied to adjust the image. A total of 80 journals were counted. Selecting a minimum threshold of 1 yielded 79 valid connection entries and clustered into 8 co-occurrence networks (Figure 6). Nodes represent journal publication volumes, while node colors denote cluster affiliations. Analysis reveals numerous journals publishing relevant articles in this field, encompassing both general-interest and cardiovascular disease-specific publications. Based on citation impact, “The European Heart Journal” (citations=224), “Journal of the American College of Cardiology” (citations=87), and “JACC-Advances” (citations=54) exhibit stronger connections with other journals. This indicates that journals like the “European Heart Journal” serve not only as key platforms for publishing significant research in this field but also occupy relatively central positions in knowledge dissemination, demonstrating considerable academic influence.

### 3.5 Highly cited publications analysis

**Figure 7:**
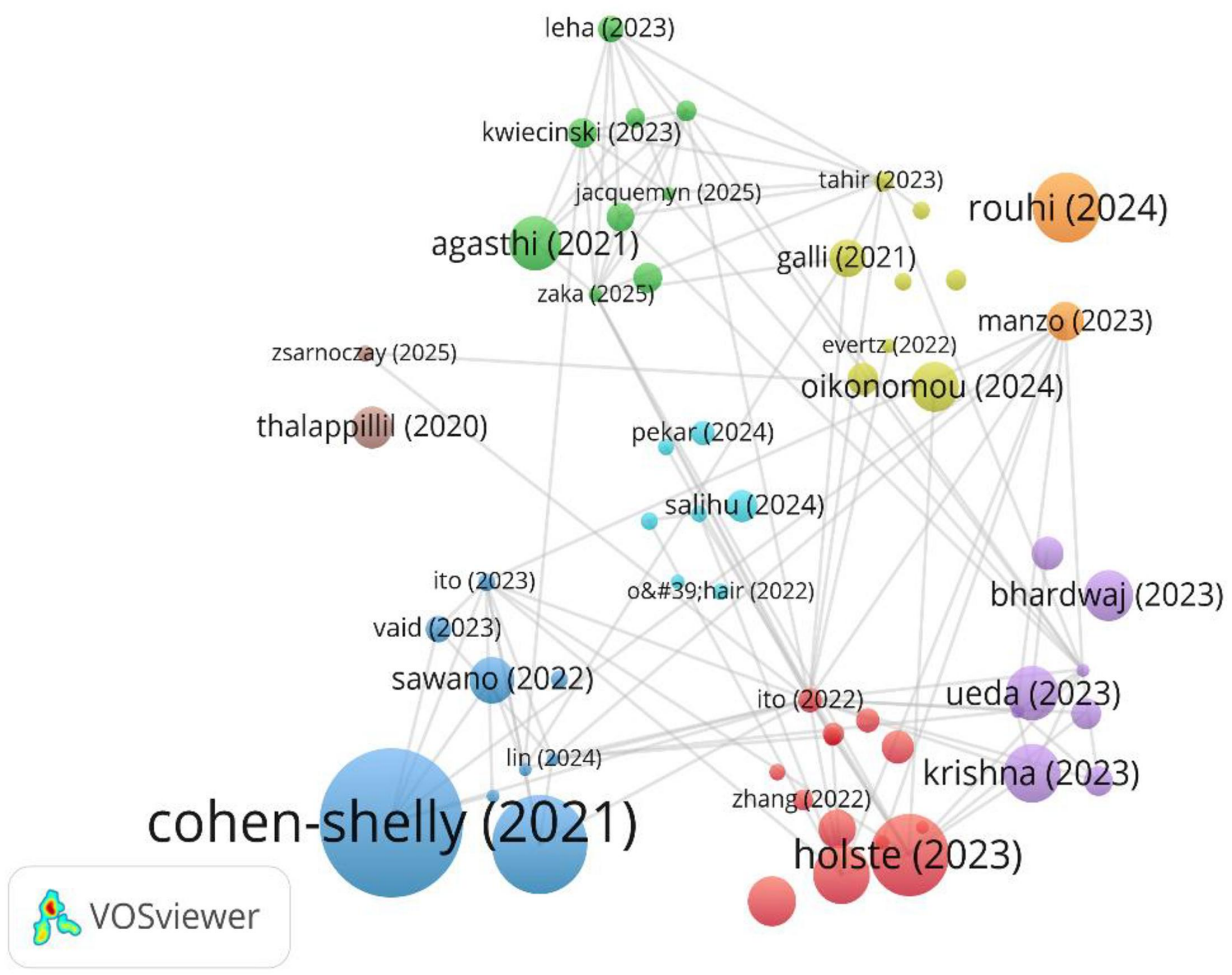
Bibliographic coupling of highly cited publications. Node size = citation frequency.

A total of 119 documents were identified using the “bibliographic coupling-Documents” module in VOSviewer software, with 59 articles having citation frequencies ≥5. Setting the minimum threshold to 5 yielded 58 valid connection entries, clustering into a visual network diagram with 8 clusters (Figure 7, Table 3). Larger nodes indicate higher citation frequencies, while node connections represent literature associations. Thicker and more numerous lines indicate documents occupying relatively central positions with higher academic value. Statistical outcome analysis revealed that Cohen-Shelly (2021) ranked first in citation frequency with 157 citations, followed by Elias (2022), Holste (2021), Rouhi (2024), and Dweck (2023) (18–22).

**Table 3:** Top 5 highly cited references on AI technology in aortic valve disease.

First Author	Years	Title	Journal	Frequency
Shelly (18)	2021	Electrocardiogram screening for aortic valve stenosis using artificial intelligence.	European Heart Journal	157
Elias (19)	2022	Deep Learning Electrocardiographic Analysis for Detection of Left-Sided Valvular Heart Disease	Journal of The American College of Cardiology	87
Holste (20)	2021	Severe aortic stenosis detection by deep learning applied to echocardiography	European Heart Journal	67
Rouhi (21)	2024	can artificial intelligence improve the readability of patient education materials on aortic stenosis? a pilot study	Cardiology And Therapy	53
Dweck (22)	2023	multi-modality imaging in aortic stenosis: an eacvi clinical consensus document	European Heart Journal-cardiovascular Imaging	42

### 3.6 Analysis of citations in references

**Figure 8:**
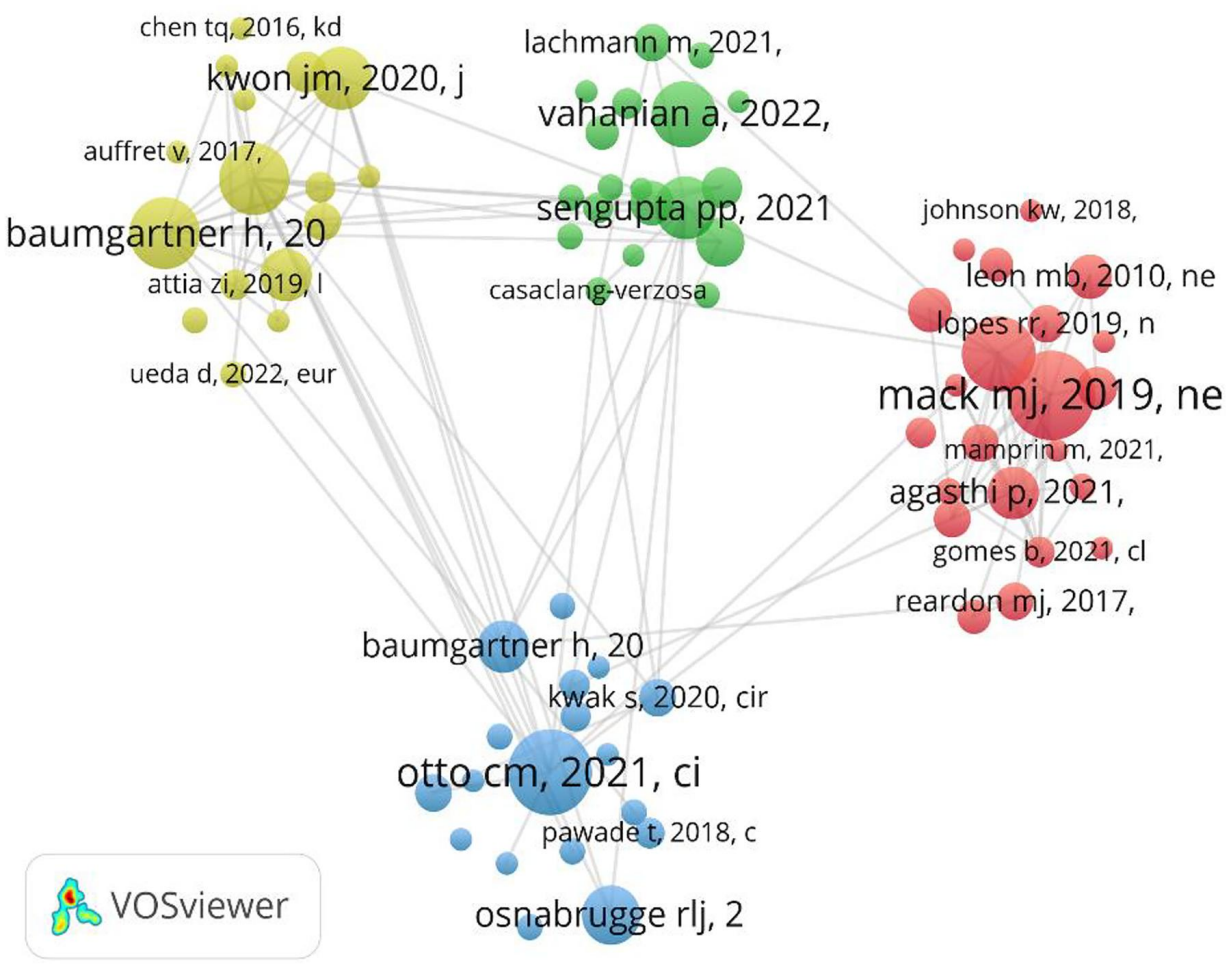
Co-occurrence map of AI technology applications in aortic valve disease research. Node size = co-citation frequency.

Using the “Co-citation-Cited References” module in VOSviewer software, we identified a total of 3,701 co-cited references, with 75 references co-cited ≥5 times. Setting the minimum threshold to 5 yielded a co-occurrence map with 75 connected entries and 4 clusters (Figure 8, Table 4). Larger nodes indicate higher co-citation frequencies, while node colors represent reference clusters. Connecting lines denote co-citation relationships between references, with thicker and more numerous lines signifying greater pioneering contributions and influence. Analysis reveals that AI technology applications in aortic valve disease generate a substantial number of co-cited references with active co-citation relationships. The most frequently co-cited literature primarily focuses on deep learning model prediction and the deep integration of AI technology with electrocardiography (23–26).

**Table 4:** Top 5 highly cited references on AI applications in aortic valve disease.

First Author	Years	Title	Journal	Frequency
Michael (23)	2019	Transcatheter Aortic-Valve Replacement with a Balloon-Expandable Valve in Low-Risk Patients	New England Journal of Medicine	24
Otto (24)	2021	2020 ACC/AHA Guideline for the Management of Patients With Valvular Heart Disease: A Report of the American College of Cardiology/American Heart Association Joint Committee on Clinical Practice Guidelines	Circulation	23
Dagmar F (25)	2019	Reply: Leveraging Machine Learning to Generate Prediction Models for Structural Valve Interventions	Jacc-cardiovascular Interventions	20
Shelly (18)	2021	Electrocardiogram screening for aortic valve stenosis using artificial intelligence.	European Heart Journal	19
Baumgartner (26)	2017	2017 ESC/EACTS Guidelines for the management of valvular heart disease	European Heart Journal	19

### 3.7 Keyword analysis

#### 3.7.1 Co-occurrence analysis

**Figure 9:**
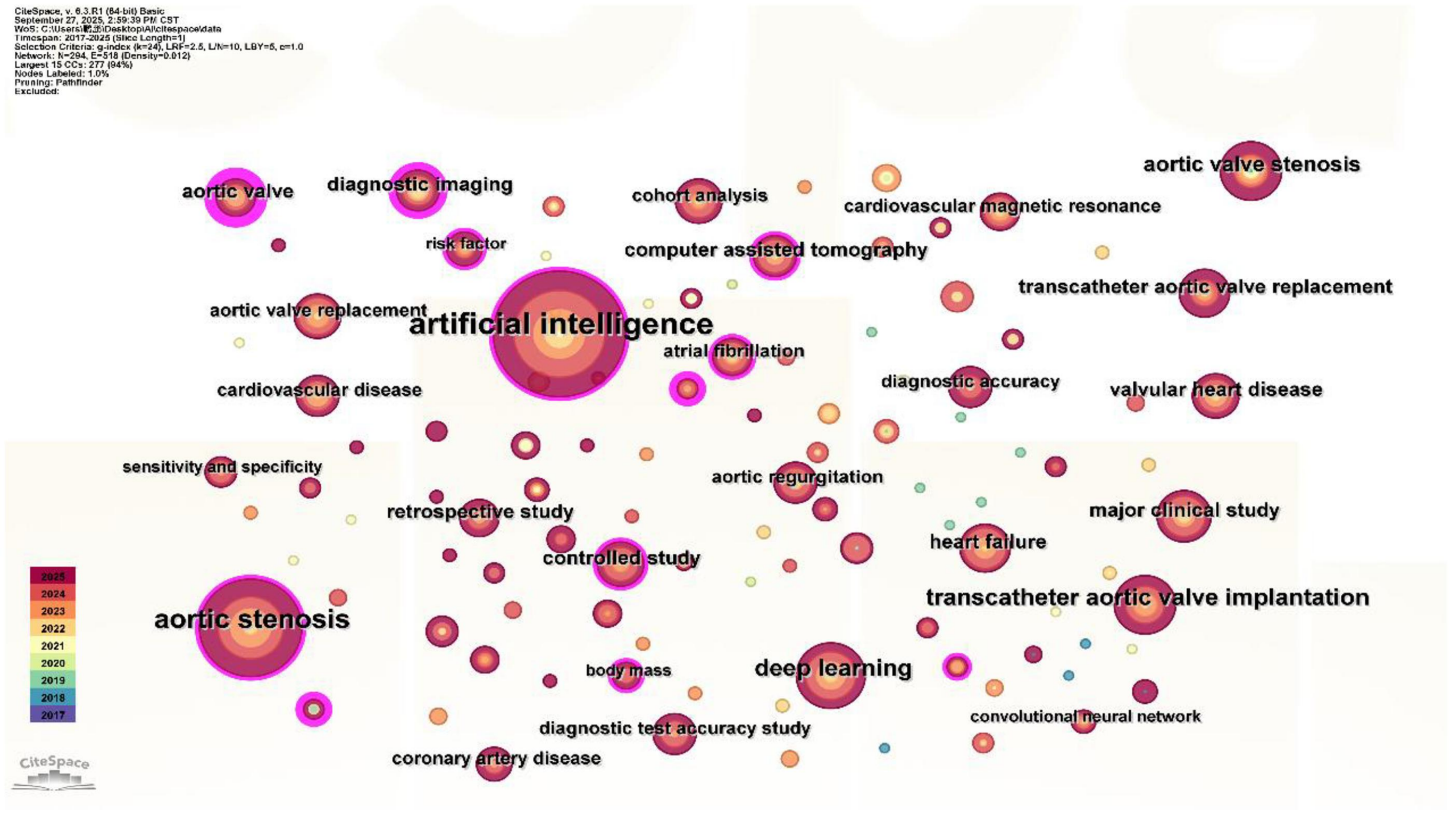
Keyword co-occurrence map of AI technology applications in aortic valve disease,. ode size = frequency; links = co-occurrence.

By extracting and analyzing high-frequency keywords in the literature, one can accurately grasp the distribution characteristics of research topics within a specific discipline and thereby conduct an in-depth analysis of its development. Selecting “keywords” as the node type, the CiteSpace software was used to construct a co-occurrence network map of literature keywords. The analysis incorporated 294 nodes and 518 connections, with a network density of 0.012 (Figure 9, Tables 5, 6).

**Table 5:** Top 10 keywords by frequency in AI applications for aortic valve disease.

Keywords	Frequency	Centrality
artificial intelligence	189	0.17
aortic stenosis	104	0.19
machine learning	82	0.01
deep learning	54	0.00
transcatheter aortic valve implantation	50	0.05
major clinical study	33	0.00
heart failure	31	0.02
diagnostic imaging	28	0.25
computer assisted tomography	25	0.17
predictors	24	0.10

**Table 6:** Top 10 keywords for AI applications in aortic valve disease management centers.

Keywords	Centrality	Frequency
aortic valve	0.42	23
80 and over	0.32	6
diagnostic imaging	0.25	28
computed tomographic angiography	0.24	11
controlled study	0.20	29
aortic stenosis	0.19	104
risk factor	0.18	15
artificial intelligence	0.17	189
computer assisted tomography	0.17	25
atrial fibrillation	0.17	19

Tables 5, 6 show the top 10 keywords ranked by frequency of occurrence and intermediary centrality. Representative keywords include artificial intelligence (189), aortic stenosis (104), and machine learning (82). After excluding terms unrelated to the retrieval strategy, the English keywords with the highest intermediary centrality were 80 and over (0.32), diagnostic imaging (0.25), and computed tomographic angiography (0.24). These results indicate that research in this field focuses on core themes such as “aortic stenosis” and “transcatheter aortic valve replacement” in content, while methodologically aligning with “computed tomographic angiography(0.24).” These results indicate that research in this field focuses on core themes such as aortic stenosis and transcatheter aortic valve replacement, with methodological ties to computed tomographic angiography and echocardiography. Table 7 presents the top 10 emerging keywords for 2025, including “heart rhythm” (2), “risk factors” (4), and “deep learning” (2). These emerging keywords reflect the field's research frontiers gradually extending toward arrhythmia-related analysis, risk factor identification, and the application of deep learning technologies.

**Table 7:** Top 10 keywords for AI applications in aortic valve disease by 2025.

Keywords	Frequency	Centrality
heart rhythm	2	0.04
risk factors	4	0.03
deep learning	2	0.02
left ventricular end-diastolic diameter	2	0.01
aortic stenosis	2	0.01
analytical parameters	2	0.01
heart ejection fraction	3	0.00
doppler echocardiography	3	0.00
risk prediction	3	0.00
4d flow MRI	2	0.00

#### 3.7.2 Cluster analysis

**Figure 10:**
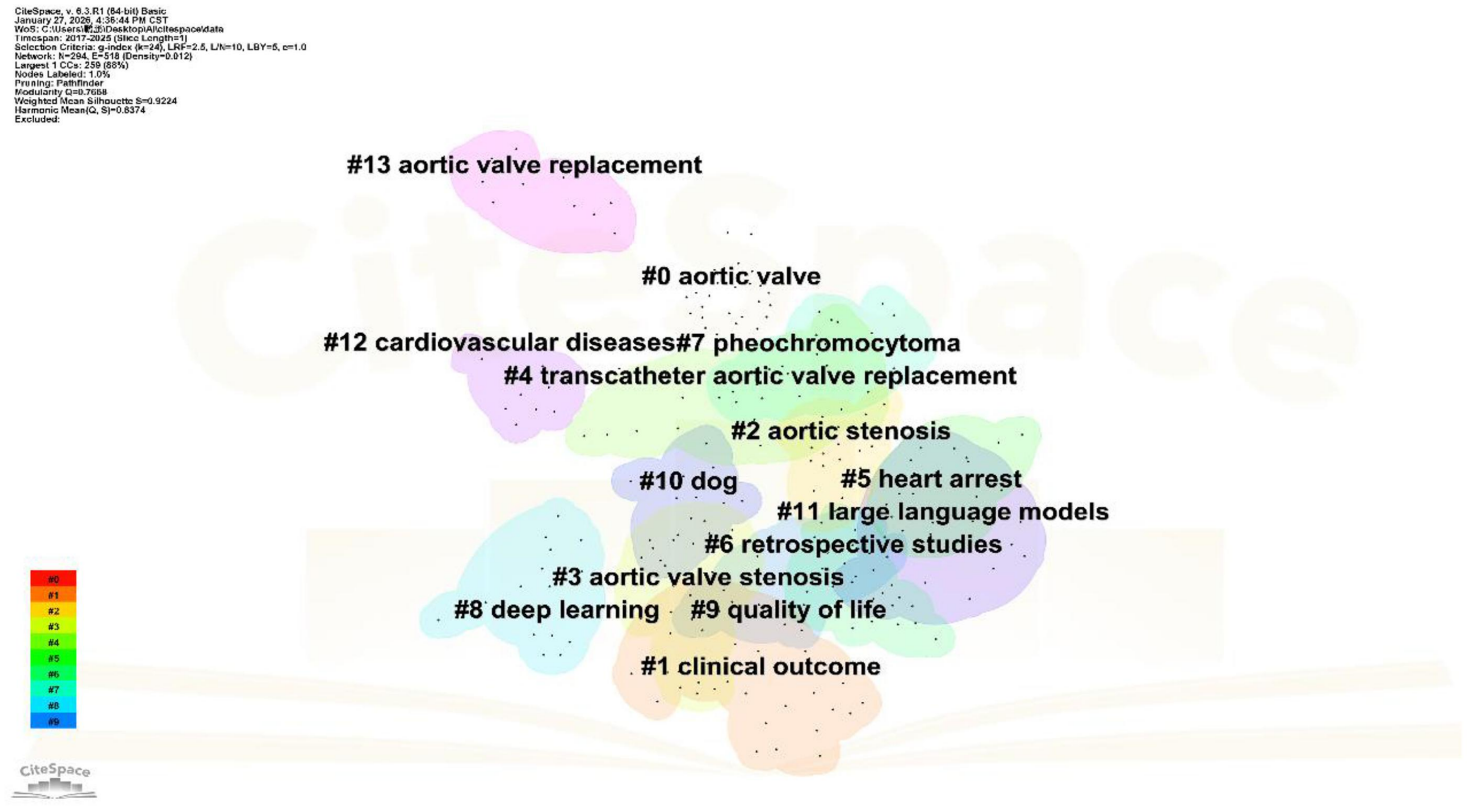
Keyword clustering map of AI technology applications in aortic valve disease research colors represent thematic clusters. Q = 0.7668, S = 0.9224.

Clustering typically relies on three algorithms: log-likelihood ratio (LLR), latent semantic indexing (LSI), and mutual information (MI). LLR is a hypothesis-testing-based text clustering method that measures whether a word (or feature) appears more frequently than expected within a cluster. Emphasizing co-occurrence significance, the LLR algorithm was selected for keyword clustering analysis. The clustering effectiveness was evaluated using the CiteSpace clustering module's Q-value and average silhouette coefficient (S-value). The analysis generated 294 nodes and 518 connections, with Q = 0.7668 (>0.3) and S = 0.9224 (>0.7). These values indicate a reasonable and highly credible clustering outcome, confirming the validity of the research keyword clusters. Analysis of the keyword clustering results using the LLR algorithm (Figure 10) reveals that AI research in aortic valve disease can be categorized into the following thematic directions: ① Disease Diagnosis and Assessment (#2, #3 Aortic Stenosis), focusing on the pathological characteristics and clinical diagnosis of the disease itself; ② Therapeutic techniques and interventions (#4, #13 Transcatheter Aortic Valve Replacement), reflecting current primary treatment modalities and research hotspots for aortic valve disease; ③ AI methods and technological applications (#8 Deep Learning, #11 Large Language Models); ④ Clinical outcomes and quality of life research (#1 Clinical Outcomes, #9 Quality of Life); ⑤ Research methodologies and data sources (#6 Retrospective Studies).

#### 3.7.3 Timeline analysis

**Figure 11:**
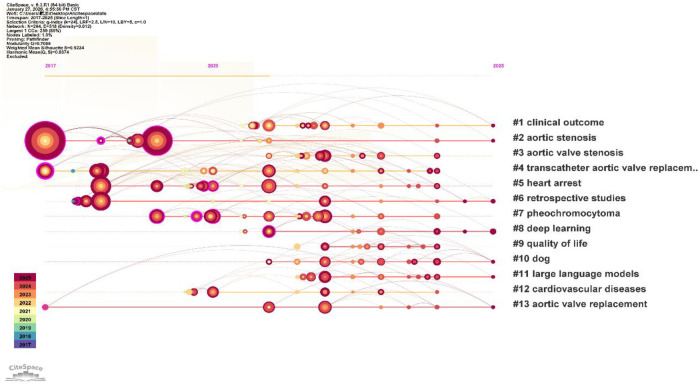
Keyword time distribution of AI technology applications in aortic valve disease. Horizontal axis = years; vertical axis = cluster IDs; node size = importance.

A timeline diagram visualizes the temporal dimension based on clustering, illustrating the dynamic evolution of different research themes (clusters) over time. This aids in predicting future trends or conducting comparative analysis across phases. By using the “timeline” module in CiteSpace software to generate keyword timeline charts, reflecting the chronological development of research themes. The vertical axis displays cluster IDs, with each horizontal band corresponding to a cluster (research theme). Each node represents a keyword, where larger nodes indicate greater importance. Connecting lines between nodes denote co-occurrence relationships among keywords (Figure 11). Research initially focused on core diseases and treatment methods, such as #2 (Aortic Stenosis), #3 (Aortic Valve Stenosis), #4 (Transcatheter Aortic Valve Replacement), and #13 (Aortic Valve Replacement). Studies on these topics remain consistently active, forming the stable structural foundation of this field. Concurrently, topics represented by #1 (clinical outcomes) and #9 (quality of life) have emerged frequently in recent years, reflecting ongoing research into the clinical translation value of AI technologies and the assessment of long-term patient prognosis. Technologically, the map reveals that methods centered on #8 (Deep Learning) emerged after 2021, becoming the core of methodological innovation. Meanwhile, #11 (Large Language Models), with activity concentrated after 2023, signifies the beginning of AI technology applications in this field.

#### 3.7.4 Keyword salience analysis

**Figure 12:**
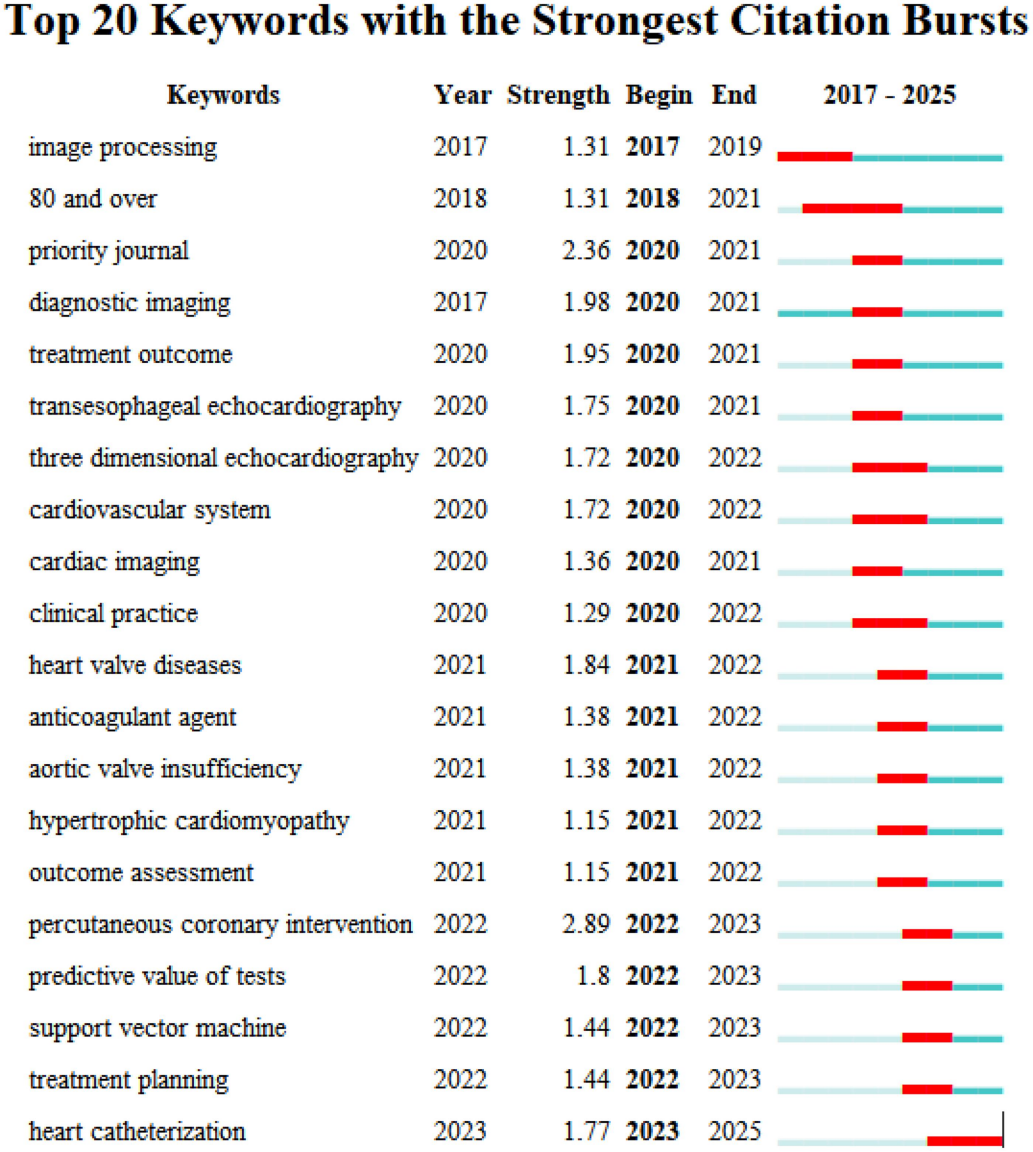
Top 20 emergent terms in AI applications for aortic valve disease. Red bars indicate burst periods.

Emerging keywords refer to terms that exhibit a significant increase in frequency over a specific period, offering a more comprehensive view of current research trends and scientific frontiers (27, 28). The “hotpot” module in CiteSpace software was used to generate emergent keyword maps. “Year” indicates the year of keyword emergence, “Strength” represents emergence intensity (higher values denote greater attention during a specific period), while ‘Begin’ and “End” denote the start and end years of emergence. Red lines indicate the duration of emergence, and blue lines indicate periods with related research but overall low activity. As shown in Figure 12, early research hotspots primarily focused on preliminary explorations of imaging diagnostics and technical methodologies. Emergent keywords such as “transesophageal echocardiography,” “three-dimensional echocardiography,” and “diagnostic imaging” indicate that research during this period concentrated on utilizing and optimizing multimodal cardiac imaging as the data foundation for AI models. Simultaneously, the emergence of keywords like “treatment outcome” and “clinical practice” reflects early studies’ emphasis on bridging clinical outcomes.

From 2021 to 2023, research shifted toward disease-specific insights and predictive model development. On one hand, the emergence of specific diseases like “aortic valve insufficiency” and “hypertrophic cardiomyopathy” signaled an expansion of research scope beyond aortic valve stenosis to other valvular diseases and comorbidities. On the other hand, the emergence of terms like “support vector machine” and “predictive value of tests” indicates a shift in focus from pure image analysis to risk prediction and diagnostic model validation based on machine learning algorithms.

The 2022–2025 period saw a deepening focus on interventional therapies and clinical integration. The sustained prominence of keywords like “percutaneous coronary intervention,” “treatment planning,” and “heart catheterization” indicates that research frontiers are converging with specific interventional treatments and perioperative management. This reflects the evolution of research hotspots in this field toward AI-assisted decision-making encompassing the entire process: diagnosis, risk assessment, treatment planning, and prognosis prediction.

## 4 Discussion

### 4.1 Research Status

This study conducts a bibliometric analysis of research on AI applications in aortic valve disease from 2016 to 2026, deconstructing its global developmental dynamics through quantitative metrics. Data reveals a sustained upward trend in annual research output within this field over the past decade. The United States holds absolute dominance in both publication volume and paper impact, with its core institutions—such as the Mayo Clinic—forming collaborative networks and knowledge dissemination channels. In contrast, other countries still exhibit a significant gap in both the scale of academic output and their pivotal influence within the knowledge network.

Additionally, this study employed VOSviewer software to conduct journal visualization analysis of the literature in this field, identifying 80 publishing journals. Among these, journals such as the “European Heart Journal” and “JAMA-Cardiology” exhibit the strongest connections with other publications, forming the core journal cluster of this field. Researchers can focus on these journals to stay abreast of field developments and identify research hotspots. Judging by the quality and standing of source journals, AI applications in aortic valve disease research demonstrate high academic value and practical potential overall. However, publications in high-impact journals remain scarce. AI-enabled, interdisciplinary medical-engineering research will persist as a hotspot, with expectations for more high-quality papers to emerge.

Co-citation network analysis of highly cited papers further reveals that research in this field primarily focuses on AI-enabled cardiac imaging and electrocardiogram analysis. Examples include AI-based screening for aortic valve stenosis via electrocardiograms and the application of deep learning for automated detection and quantitative analysis of aortic valve lesions in echocardiography. These studies form the methodological and clinical validation foundation of the field. In terms of journal distribution, these high-impact publications predominantly appear in leading international cardiovascular journals such as “European Heart Journal”, “Journal of the American College of Cardiology”, reflecting that the outcomes of this interdisciplinary field have gained acceptance on high-level academic platforms.

The sharp rise in publications after 2020 may reveal that the maturation of deep learning techniques, particularly convolutional neural networks, enabled automated feature extraction from medical images. Second, the growing availability of large-scale echocardiographic and CT databases provided researchers with sufficient labeled data to train and validate models and regulatory approvals of AI tools in cardiovascular medicine created a favorable environment for clinical translation.

The timeline analysis reveals three distinct phases. From 2016 to 2019, studies focused on imaging-based AI, applying traditional machine learning to echocardiographic and CT features. Between 2020 and 2023, deep learning enabled end-to-end detection of aortic stenosis and regurgitation from raw images. Since 2024, the field has shifted toward multimodal and predictive models, integrating clinical data, ECG, and imaging for risk stratification, with large language models emerging for patient education. The shift from monomodal imaging to multimodal prediction reflects the field's growing clinical focus.

However, This concentration of research funding within a few well-funded institutions and national agencies risks knowledge monopolies and limited research diversity. Future funding should incentivize multi-center, international collaborations to ensure globally representative datasets.

### 4.2 Conceptual mapping of AI research themes onto clinical stages

**Figure 13:**
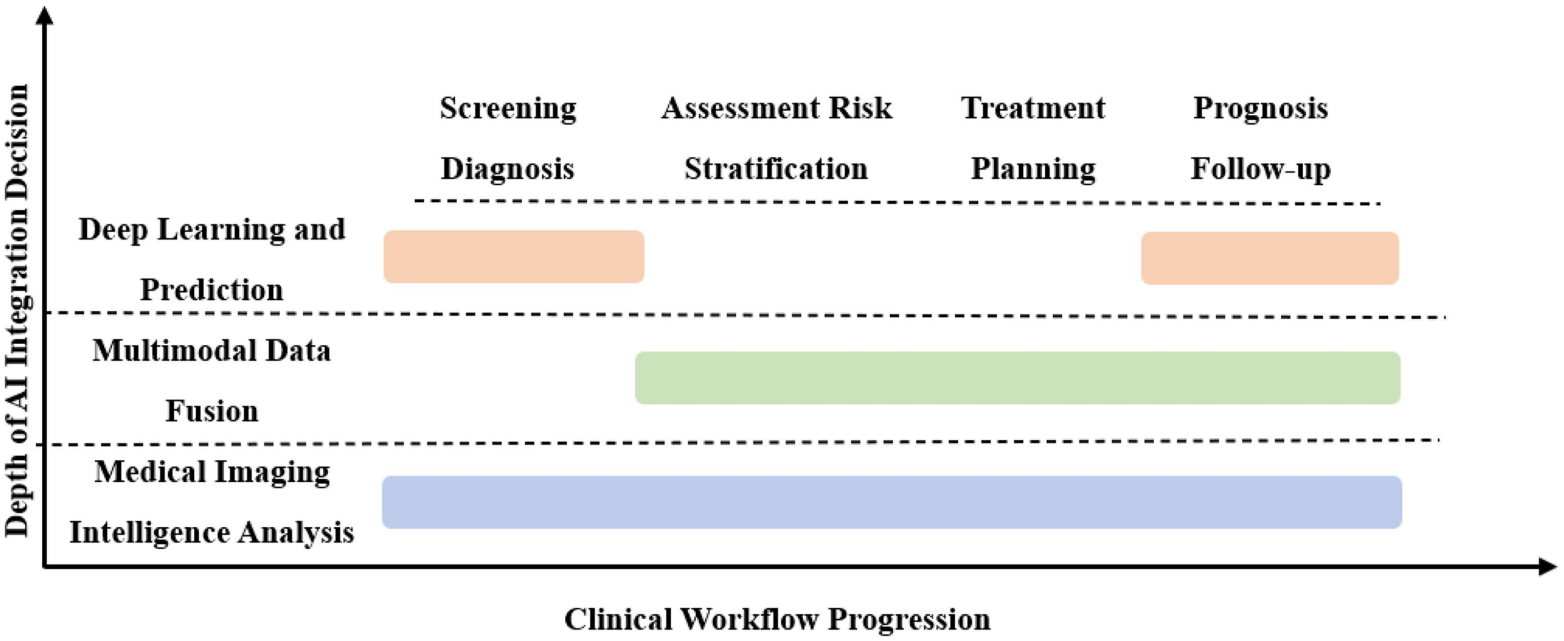
Framework of AI technology in the diagnosis and treatment of aortic valve disease. Conceptual mapping of AI research themes onto clinical stages of aortic valve disease management. This framework is derived from keyword clustering patterns and is intended as a hypothesis-generating schematic, not a validated clinical pathway.

Based on the aforementioned scientometric analysis, utilizing keyword co-occurrence and clustering analysis (Figures 9, 10), combined with the timeline (Figure 11) and emergent terms (Figure 12), and adhering to standard clinical management guidelines for aortic valve diseases (24, 26), we propose a “conceptual synthesis” that maps the identified research themes onto clinical stages. (Figure 13). This mapping aims to systematically deconstruct the AI technology-enabled disease management process. Figure 13 indicates: (1) Intelligent medical imaging analysis (deep learning) serves as a foundational tool throughout the entire diagnostic and therapeutic process, providing a basis for automated measurement and quantitative assessment; (2) Multimodal data fusion and prediction become central to risk assessment, treatment selection, and risk forecasting after obtaining an initial diagnosis; (3) Natural language processing and patient management appear at both the beginning and end of the process—assisting in interpreting diagnostic reports early on and involving patient education and long-term follow-up management later.

#### 4.2.1 Application of AI technology in aortic valve stenosis

Keywords encapsulate the core content of literature, and keyword analysis can directly reflect research themes and hotspots (29). Combining co-occurrence and clustering results analysis, aortic valve stenosis (Count: 104, Centrality: 0.19) emerged as a high-frequency keyword. AS is one of the most common valvular heart diseases (30). As a chronic progressive disease, its pathological features include lipid accumulation, inflammation, fibrosis, and calcification. The left ventricle adapts to increased afterload by developing concentric hypertrophy, leading to diastolic dysfunction. Its progression is insidious, and the emergence of major clinical symptoms such as angina, syncope, and heart failure often indicates poor prognosis (31, 32). With advancing population aging, the incidence of AS increases annually. In Western countries, the prevalence of aortic valve stenosis in individuals over 75 years old is 2.8%, ranking third after hypertension and coronary heart disease (33). Conservative management yields poor outcomes for severe AS patients. Improving prognosis primarily relies on identifying high-risk patients, assessing stenosis severity, managing comorbidities, and determining the timing and type of aortic valve replacement.

In the realm of early diagnosis and screening, artificial intelligence has charted new territory for automated heart sound recognition. The Shelly (18) team investigated the use of artificial intelligence-enabled electrocardiogram (AI-ECG) to identify patients with moderate to severe aortic stenosis. Their findings revealed that patients with AI-ECG false positives had a twofold higher risk of developing moderate to severe AS within 15 years compared to those with negative AI-ECG results, (hazard ratio 2.18, 95% confidence interval 1.90–2.50). The Holste team developed a three-dimensional convolutional neural network for detecting aortic stenosis. This neural network model was validated across 2,040 consecutive studies and can provide interpretable results by highlighting the aortic valve, mitral annulus, and left atrium as predictive regions. It demonstrated potential utility in screening for non-severe AS cases (20).

Severity Assessment and Risk Stratification: Aortic valve calcification is an effective predictor of AS severity (34). Pandey quantified aortic valve calcium content in AS patients using computed tomography angiography (CTA) and evaluated it with semi-automated coronary assessment software. Their findings demonstrated that valve calcification measurements on CTA exhibit high accuracy and warrant broader adoption (35). Slostad employed pixel density quantification software to objectively measure valve calcification via two-dimensional echocardiography for AS severity differentiation. Results indicated that the two-dimensional echocardiography-quantitative software method serves as an effective adjunct for predicting AS severity (36); Garin's team employed AI technology for automated surgical risk stratification in patients scheduled for TAVR. Results demonstrated that this automated analysis process achieved over 90% consistency with multidisciplinary cardiac team decisions (37). It should be noted that while these preliminary studies demonstrate AI's potential in automated quantitative assessment, existing evidence primarily focuses on validating technical feasibility (38, 39) and lacks large-scale, prospective clinical studies to confirm its advantages. This limitation restricts the widespread clinical application of relevant AI technologies. Therefore, AI-driven automated quantitative assessment methods represent a promising direction for future exploration.

In prognosis prediction and follow-up management, machine learning-based prognostic models are emerging as crucial tools for risk stratification (40). By integrating preoperative imaging features, intraoperative data, and early postoperative recovery indicators, AI models can dynamically assess patients’ long-term mortality, readmission rates, and cardiac function recovery (40, 41). The emergence of the sudden-onset words “risk factors” and “risk prediction” in this study reflects the research momentum in this direction.

On the other hand, technologies such as artificial intelligence and large language models are being applied in patient education, follow-up communication, and health management. Research by Rouhi (21) has explored using AI to optimize the readability of educational materials for patients with aortic stenosis. Future potential hotspots may include follow-up systems based on large language models and AI algorithms for arrhythmia detection in wearable devices (heart rhythm, Centrality: 0.04), enabling real-time home monitoring of complications such as atrial fibrillation following TAVR.

#### 4.2.2 Application of AI technology in aortic valve regurgitation

Compared to aortic stenosis, AI research in aortic regurgitation remains at an early stage. In our keyword co-occurrence analysis, “aortic regurgitation” appeared only 23 times (centrality 0.01) and did not form an independent cluster, indicating a fragmented and less mature research landscape. This is consistent with the clinical reality: AR pathophysiological quantification is more complex than AS, relying on multiple parameters (regurgitant volume, effective regurgitant orifice area, pressure half-time) and lacking a single robust imaging biomarker (42). The China-VHD Registry Study indicates that isolated AR accounts for the highest proportion (38.8%) among patients with moderate-to-severe aortic valve disease in China (43). Degenerative disease is the primary cause, with other etiologies including infection, rheumatic endocarditis, aortic dissection, and syphilis (44). Clinically, transthoracic echocardiography (TTE), cardiac CT, and MRI can assess AR severity and perform risk stratification, enabling early treatment planning for patients.

Current AI efforts are primarily focused on screening and preliminary detection. Guo's team (45) conducted research on deep learning-based automated echocardiographic detection of aortic regurgitation. They developed cross-sectional classification and valve regurgitation recognition models using deep learning algorithms and analyzed their performance. Results demonstrated that deep learning algorithms can automatically identify valve regurgitation, showing potential as a screening tool for valvular heart disease. Another study published in the “European Heart Journal” found that an AI-ECG model developed by the team can guide echocardiographic monitoring for patients with aortic regurgitation, facilitating early detection and treatment of the condition. This model has been validated across multiple countries and diverse ethnic cohorts (46).

Disease Progression Prediction and Risk Stratification: Unlike AS, the assessment of AR severity and disease progression prediction relies on comprehensive multi-parameter quantification. Although Guo (45) demonstrated the feasibility of AI-based automatic detection, advancing beyond detection to precisely quantify regurgitant volume and effective regurgitant orifice area remains a significant bottleneck. Long developed an AI system for assessing valve regurgitation and risk stratification. Their study demonstrated the system's ability to accurately classify and predict aortic regurgitation, mitral regurgitation, and disease progression risk. However, this AI system exhibited low specificity for AS (47). Malahfji's team (48) employed machine learning to identify distinct phenotypic groups among AR patients and evaluate their prognosis. They initially hypothesized AR patients as a homogeneous population, yet clinically observed varying disease progression and outcomes. Subsequently, patients underwent CMR examinations, and machine learning methods were applied to describe AR patient phenotypes using CMR outcomes and clinical characteristics. This approach may benefit AR patient risk stratification. Currently, most AI models still struggle to accurately segment regurgitant flow from cardiac magnetic resonance (CMR) images. Therefore, establishing a large-scale, high-quality database of dynamic AR imaging is crucial.

Current evidence indicates that the application of AI technology in AR management is primarily focused on screening, preliminary identification, and risk stratification. Substantive research in areas such as assessing the stability of non-calcified areas and predicting valve displacement risks remains significantly less developed compared to AS. Additionally, AI-driven personalized education and management platforms for AR patients represent a promising future research direction. AI has shown promise in general cardiovascular imaging and arrhythmia monitoring (49). However, specific applications in AR remain limited, and future efforts should prioritize the establishment of open-access annotated AR imaging databases. Based on this, this study recommends establishing a standardized, open-access AR imaging and clinical database to provide a foundation for AI algorithms. Concurrently, research investment in dynamic hemodynamic assessment and ventricular remodeling prediction for AR is essential to better leverage AI technology in AR management.

### 4.3 Comparison with broader AI applications in cardiovascular medicine

In coronary artery disease, AI models for plaque characterization and ischemia detection have achieved clinical integration, supported by large annotated datasets from multicenter trials and active industry engagement (50). In heart failure, AI-enhanced electrocardiography and digital twin models are increasingly incorporated into clinical workflows for early diagnosis and remote monitoring (8).

Compared to these fields, AI in aortic valve disease faces challenges. First, data availability is more limited: although large echocardiography databases exist, few are publicly accessible with pixel-level annotations for valve-specific structures such as leaflet calcification or orifice area (51). Second, industry participation remains lower, with fewer commercial products targeting valve disease compared to coronary or heart failure applications.

### 4.4 Clinical readiness and implementation challenges

Despite growing research output, the clinical readiness of AI in aortic valve disease remains low. Most studies are retrospective and single-center; prospective, multicenter validation is lacking for almost all models (18, 20).

Generalizability is another concern. Training datasets typically originate from high-volume academic centers, raising the risk of performance drift when applied to different populations, imaging devices, or community settings (46). External validation in diverse real-world environments is urgently needed.

Implementation barriers include poor integration with existing clinical workflows, clinician distrust of algorithms. Addressing these challenges will require prospective trials, open benchmarking datasets, and explainable AI methods.

### 4.5 Future directions

Three emerging themes from our analysis warrant prioritized research. First, deep learning should advance from retrospective detection to prospective validation of automated severity grading, particularly for aortic stenosis quantification. Second, large language models offer opportunities for patient Rehabilitation Education and clinical documentation. Third, multimodal data integration, combining imaging, ECG, and biomarkers, can improve risk stratification and timing of intervention in asymptomatic severe stenosis. Future efforts should focus on prospective multicenter trials, LLM-based communication tools, and open-access multimodal datasets linking imaging with longitudinal outcomes.

## 5 Limitations

This study has limitations. First, the literature search was limited to the Web of Science Core Collection and Scopus databases, which may have omitted relevant studies indexed elsewhere. Second, only English-language publications were included, potentially introducing language bias. Third, bibliometric methods primarily quantify publication patterns and citations, which may not fully capture the clinical impact or quality of individual studies. Additionally, the use of CiteSpace and VOSviewer involves parameter selection that may influence network visualization outcomes; however, we used default settings to ensure reproducibility and Parameter choices may affect minor details but not the main conclusions.

## 6 Conclusion

This study employs scientometric analysis using CiteSpace and VOSviewer to deconstruct the knowledge structure, collaborative networks, and evolutionary trends of artificial intelligence applications in the field of aortic valve diseases (2016–2026).The analysis reveals rapid growth in research output within this domain, forming a global landscape centered on the United States with strong institutional collaboration. Concurrently, the study reveals significant application disparities between aortic stenosis and aortic regurgitation. Future research priorities include deepening multimodal data fusion and AI model development, alongside expanding AI applications in patient health management. This work establishes a foundational knowledge base for understanding developments in this interdisciplinary field.

## Data Availability

The datasets presented in this study can be found in online repositories. The names of the repository/repositories and accession number(s) can be found in the article/Supplementary Material.
