# Supplementary material for: Artificial intelligence technology in aortic valve disease: a decade of scientometric and narrative review
Source: Front Cardiovasc Med. 2026 Jul 9;13:1843658. doi: 10.3389/fcvm.2026.1843658 (PMC13391551; doi:10.3389/fcvm.2026.1843658)
Supplement: Supplementary file 1 [file Datasheet1.docx]

The WoSCC search query is:

#1 “Artificial Intelligence” OR “Intelligence, Artificial” OR “Computer Reasoning” OR “Reasoning, Computer” OR “AI (Artificial Intelligence)” OR “Machine Intelligence” OR “Intelligence, Machine” OR “Computational Intelligence” OR “Intelligence, Computational” OR “Computer Vision Systems” OR “Computer Vision System” OR “System, Computer Vision” OR “Systems, Computer Vision” OR “Vision System, Computer” OR “Vision Systems, Computer” OR “Knowledge Acquisition (Computer)” OR “Acquisition, Knowledge (Computer)” OR “Knowledge Representation (Computer)” OR “Knowledge Representations (Computer)” OR “Representation, Knowledge (Computer)”

#2 “Aortic Valve” OR “Aortic Valves” OR “Valve, Aortic” OR “Valves, Aortic”

#3 “Aortic Valve Stenosis” OR “Aortic Valve Stenoses” OR “Stenoses, Aortic Valve” OR “Stenosis, Aortic Valve” OR “Valve Stenoses, Aortic” OR “Valve Stenosis, Aortic” OR “Aortic Stenosis” OR “Stenoses, Aortic” OR “Stenosis, Aortic”

#4 “Aortic Valve Insufficiency” OR “Insufficiency, Aortic Valve” OR “Aortic Incompetence” OR “Incompetence, Aortic” OR “Aortic Valve Incompetence” OR “Incompetence, Aortic Valve” OR “Regurgitation, Aortic Valve” OR “Aortic Regurgitation” OR “Regurgitation, Aortic”

#5“Transcatheter Aortic Valve Replacement”OR “TAVR” OR “Transcatheter Aortic Valve Implantation”

#1 AND (#2 OR #3 OR #4 OR #5)

Scopus databases:

#1 ‘Artificial Intelligence’:ab,kw,ti OR ‘Intelligence, Artificial’:ab,kw,ti OR ‘Computer Reasoning’:ab,kw,ti OR ‘Reasoning, Computer’:ab,kw,ti OR ‘AI (Artificial Intelligence)’:ab,kw,ti OR ‘Machine Intelligence’:ab,kw,ti OR ‘Intelligence, Machine’:ab,kw,ti ‘Computational Intelligence’:ab,kw,ti OR ‘Intelligence, Computational’:ab,kw,ti OR ‘Computer Vision Systems’:ab,kw,ti OR ‘Computer Vision System’:ab,kw,ti OR ‘System, Computer Vision’:ab,kw,ti OR ‘Systems, Computer Vision’:ab,kw,ti OR ‘Vision System, Computer’:ab,kw,ti OR ‘Vision Systems, Computer’:ab,kw,ti OR ‘Knowledge Acquisition (Computer)’:ab,kw,ti OR ‘Acquisition, Knowledge (Computer)’:ab,kw,ti OR ‘Knowledge Representation (Computer)’:ab,kw,ti OR ‘Knowledge Representations (Computer)’:ab,kw,ti OR ‘Representation, Knowledge (Computer) ’:ab,kw,ti

#2 ‘Aortic Valve’:ab,kw,ti OR ‘Aortic Valves’:ab,kw,ti OR ‘Valve, Aortic’:ab,kw,ti OR ‘Valves, Aortic’:ab,kw,ti

#3 ‘Aortic Valve Stenosis’:ab,kw,ti OR ‘Aortic Valve Stenoses’:ab,kw,ti OR ‘Stenoses, Aortic Valve’:ab,kw,ti OR ‘Stenosis, Aortic Valve’:ab,kw,ti OR ‘Valve Stenoses, Aortic’:ab,kw,ti OR ‘Valve Stenosis, Aortic’:ab,kw,ti OR ‘Aortic Stenosis’:ab,kw,ti ‘Stenoses, Aortic’:ab,kw,ti OR ‘Stenosis, Aortic’:ab,kw,ti

#4 ‘Aortic Valve Insufficiency’:ab,kw,ti OR ‘Insufficiency, Aortic Valve ’:ab,kw,ti OR ‘Aortic Incompetence’:ab,kw,ti OR ‘Incompetence, Aortic’:ab,kw,ti OR ‘Aortic Valve Incompetence’:ab,kw,ti OR ‘Incompetence, Aortic Valve’:ab,kw,ti OR ‘Regurgitation, Aortic Valve’:ab,kw,ti ‘Aortic Regurgitation’:ab,kw,ti OR ‘Regurgitation, Aortic’:ab,kw,ti

#5 ‘Transcatheter Aortic Valve Replacement’:ab,kw,ti OR ‘TAVR’:ab,kw,ti OR ‘Transcatheter Aortic Valve Implantation’:ab,kw,ti

The deadline is January 10, 2026.

#1 AND (#2 OR #3 OR #4 OR #5)
